# Synthesis of High *χ*–Low *N* Diblock Copolymers by Polymerization‐Induced Self‐Assembly

**DOI:** 10.1002/anie.202001436

**Published:** 2020-05-04

**Authors:** James Jennings, Erik J. Cornel, Matthew J. Derry, Deborah L. Beattie, Matthew J. Rymaruk, Oliver J. Deane, Anthony J. Ryan, Steven P. Armes

**Affiliations:** ^1^ Dainton Building Department of Chemistry The University of Sheffield Sheffield S3 7HF UK; ^2^ Present address: Aston Institute of Materials Research (AIMR) Aston University Birmingham B4 7ET UK

**Keywords:** block copolymers, nanolithography, nanoparticle processing, polymerization-induced self-assembly, solid-state morphology

## Abstract

Polymerization‐induced self‐assembly (PISA) enables the scalable synthesis of functional block copolymer nanoparticles with various morphologies. Herein we exploit this versatile technique to produce so‐called “high *χ*–low *N*” diblock copolymers that undergo nanoscale phase separation in the solid state to produce sub‐10 nm surface features. By varying the degree of polymerization of the stabilizer and core‐forming blocks, PISA provides rapid access to a wide range of diblock copolymers, and enables fundamental thermodynamic parameters to be determined. In addition, the pre‐organization of copolymer chains within sterically‐stabilized nanoparticles that occurs during PISA leads to enhanced phase separation relative to that achieved using solution‐cast molecularly‐dissolved copolymer chains.

## Graphical abstract

**Polymerization‐induced self‐assembly** enables the scalable synthesis of functional block copolymer nanoparticles with various morphologies. This versatile technique is exploited to prepare so‐called “high *χ*–low *N*” diblock copolymers that undergo nanoscale phase separation in the solid state to produce sub‐10 nm domains.

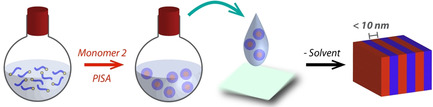

Well‐ordered polymeric materials possessing periodic domains with a characteristic length scale of less than 10 nm are attractive scaffolds for a wide range of applications. For example, nanostructured etch masks for lithography provide access to higher domain densities and hence enhanced performance for microchip technology,^1^ while microporous polymeric membranes offer considerable potential for water purification via nanofiltration.^2^ However, top‐down lithographic approaches, such as the use of extremely short ultraviolet wavelengths, become increasingly energy‐intensive when targeting sub‐20 nm patterns. In principle, block copolymer self‐assembly offers a robust route to obtain periodic nanostructures with appropriate mechanical properties and chemical functionalities, which can serve as templates for patterned or nanoporous materials using a bottom‐up approach.^3^ Clearly, shorter diblock copolymer chains are required to produce smaller domains but it is well‐known that lower molecular weight precursors have less propensity to form well‐ordered nanostructures in the solid state. This is because microphase separation requires the product of the Flory–Huggins interaction parameter (*χ*) and the mean degree of polymerization (*N*) to exceed a certain minimum value (i.e. *χN*>10.5).^4^ Fortunately, this problem can be mitigated by designing diblock copolymers with a sufficiently high interaction parameter: this has led to the emergence of a new class of so‐called “high *χ*–low *N*” diblock copolymers. These systems typically combine blocks containing heteroatoms (particularly silicon or fluorine) in either the backbone,^5^ side‐chains,^6^ or end‐groups.^7^ Alternatively, post‐polymerization modification of commodity polymers has been explored.^8^ However, most literature examples of high *χ*–low *N* block copolymers require multi‐step syntheses and extensive purification to achieve such microphase‐separated materials, making them much less cost‐effective.^9^ Moreover, subsequent processing to prepare either bulk materials or thin films usually requires extensive use of undesirable volatile organic compounds (VOCs) as processing aids.

Over the past decade or so, polymerization‐induced self‐assembly (PISA) has become widely recognized as a powerful platform technology for the rational design of a broad range of block copolymers in the form of sterically‐stabilized nanoparticles (typically spheres, worms or vesicles) in either polar or non‐polar media.^10^ In particular, reversible addition‐fragmentation chain transfer (RAFT) polymerization has enabled the efficient synthesis of functionally diverse block copolymers in environmentally‐benign solvents (e.g. water or lower alcohols) using either emulsion or dispersion polymerization techniques.10a, 10g Moreover, in some cases such PISA formulations enable one‐pot syntheses via sequential monomer addition.^11^ However, the solid‐state properties of PISA‐derived block copolymers (Scheme 1) has perhaps surprisingly remained hitherto underexplored.^12^

**Scheme 1:**
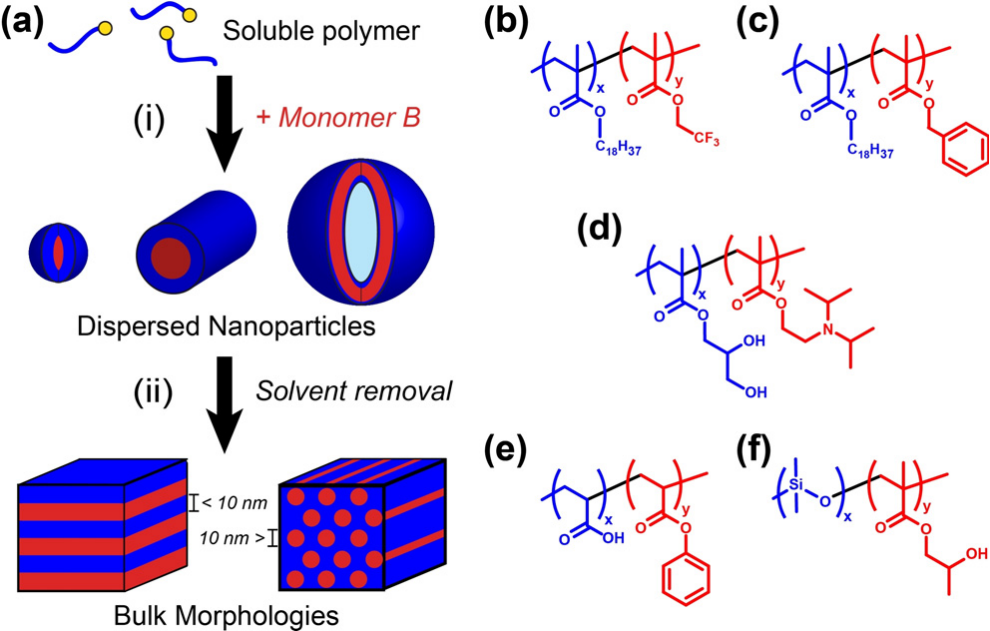
a) Schematic representation of (i) the preparation of a concentrated dispersion of AB diblock copolymer nano‐objects via polymerization‐induced self‐assembly (PISA) and (ii) their transformation into bulk nanostructures following solvent removal. Five diblock copolymers examined in the present study include: b) PSMA–PTFEMA, c) PSMA–PBzMA, d) PGMA–PDPA, e) PAA–PPhA and f) PDMS–PHPMA.

In this article, the RAFT emulsion or dispersion polymerization of commercially‐available vinyl monomers is utilized to produce a wide range of high *χ*–low *N* diblock copolymers in the form of sterically‐stabilized nanoparticles. Targeting chemically dissimilar, enthalpically incompatible blocks enables access to copolymer chains that undergo microphase separation in the bulk (or within thin films) to afford well‐defined morphologies with sub‐10 nm features. We hypothesized that the pre‐organization of the copolymer chains achieved during PISA should aid the formation of solid‐state structures after solvent removal, thus conferring processing advantages.

**Scoping phase behaviour**. The versatility of RAFT‐mediated PISA enables the facile synthesis of a wide range of AB diblock copolymers with variable relative block volume fractions.10d, 10e, 10f For proof‐of‐concept studies, poly(stearyl methacrylate) (PSMA_11_, where the subscript indicates its mean degree of polymerization, or DP, calculated by ^1^H NMR analysis) was chain‐extended via RAFT dispersion polymerization of 2,2,2‐trifluoroethyl methacrylate (TFEMA) in *n*‐tetradecane at 90 °C, as recently reported by Cornel and co‐workers.^13^ More than 95 % TFEMA conversion was obtained in such PISA syntheses, yielding a series of five PSMA_11_–PTFEMA_y_ (Scheme 1 b) nanoparticle dispersions for which *y*=9 to 48. GPC analysis indicated high blocking efficiencies and relatively low dispersities (see Table S1 and Figure S1).

These PSMA_11_–PTFEMA_y_ diblock copolymer nanoparticles were isolated by precipitation into ethanol, which is a non‐solvent for both PSMA and PTFEMA. This copolymer series exhibited a range of solid‐state morphologies, which were studied as a function of temperature using small‐angle X‐ray scattering (SAXS), see Figure 1 a. PSMA_11_–PTFEMA_9_ and PSMA_11_–PTFEMA_18_ lacked long‐range order at all temperatures investigated, as indicated by their relatively broad, ill‐defined structure factor peaks. Clearly, these two copolymers do not meet the essential criterion required for the onset of microphase separation (i.e. *χN*<10.5). On the other hand, PSMA_11_–PTFEMA_28_ formed well‐defined hexagonally‐packed cylinders (*C*), as indicated by its Bragg peaks at *q*/q*=$\sqrt{3}$ , $\sqrt{4}$ and $\sqrt{7}$ relative to the principal scattering peak at q*=0.0565 Å^−1^. PSMA_11_–PTFEMA_39_ also formed hexagonally‐packed cylinders below 130 °C (q*=0.0478 Å^−1^). However, heating this copolymer to 135 °C produced a series of additional peaks that could not be assigned to the hexagonal phase. Further heating up to 166 °C led to disappearance of the hexagonal phase, leaving a series of peaks with relative *q* positions at $\sqrt{6}$ , $\sqrt{8}$ , $\sqrt{14}$ , $\sqrt{22}$ and $\sqrt{24}$ that correspond to a double gyroid (*G*) morphology^14^ (see Figures S2 and S3 for the corresponding SAXS patterns and peak assignments). This transition was found to be reversible on cooling, and such a hexagonal‐to‐gyroid phase transition has been previously reported.^15^ Finally, a series of equally‐spaced Bragg peaks at *q*/q*=1, 2, 3 (where q*=0.0505 Å^−1^) indicated a pure lamellar (L) phase for the PSMA_11_–PTFEMA_49_ diblock copolymer at 90 °C.

**Figure 1:**
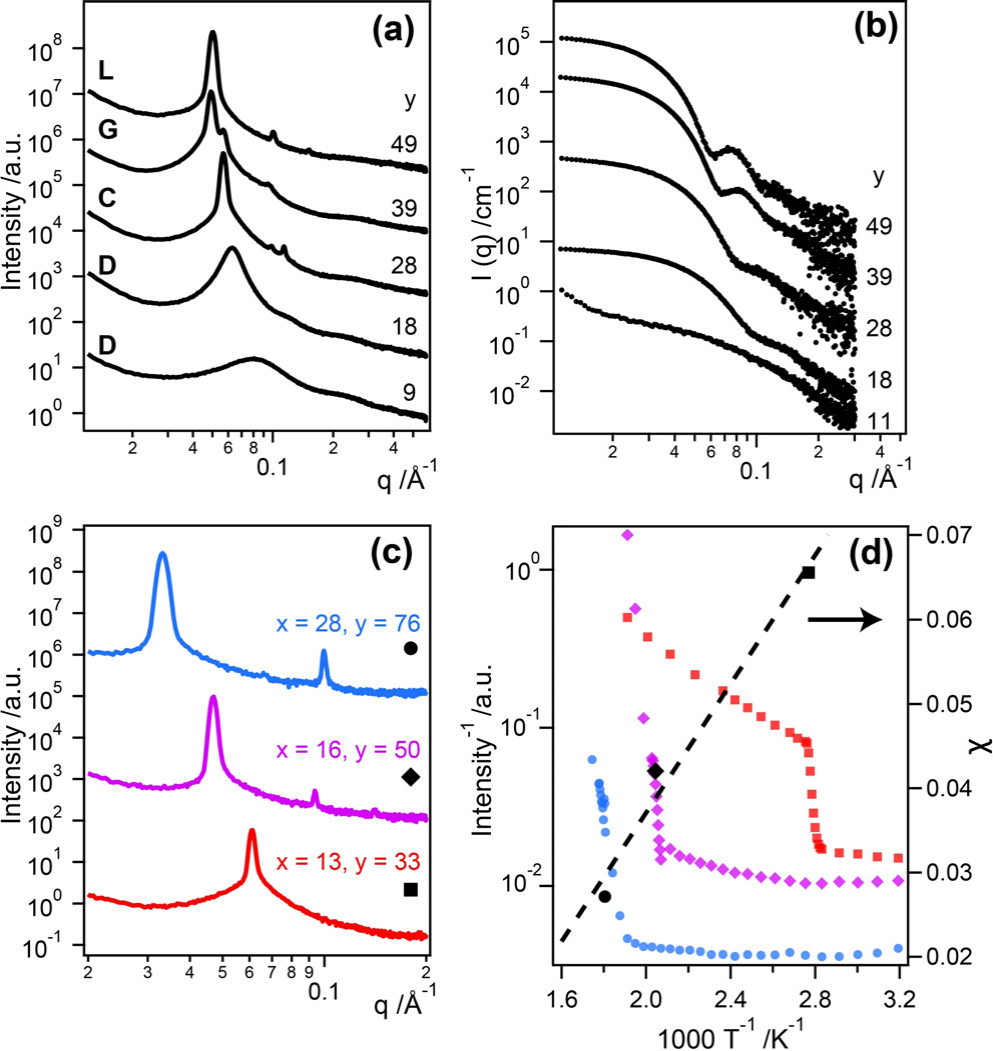
PISA can be used to screen for block copolymer phase behavior. SAXS patterns recorded for PSMA_11_–PTFEMA_y_ diblock copolymers a) in the bulk at 70 °C (*y*=18), 80 °C (*y*=9), 90 °C (*y*=28 and 49) or 166 °C (*y*=39), and b) as sterically‐stabilized nanoparticles [for 1.0 % w/w dispersions in *n*‐tetradecane] at 25 °C. Tuning the mean degree of polymerization (*y*) of the PTFEMA block provides convenient access to hexagonal (*y*=28), gyroid (*y*=39), and lamellar (*y*=49) copolymer morphologies. c) SAXS data recorded in the bulk at 80 °C for PSMA_*x*_–PBzMA_*y*_ copolymers with approximately equal block volume fractions; each copolymer exhibits a lamellar morphology with molecular weight‐dependent domain spacings. d) Order–disorder transition (ODT) temperatures (black symbols) were determined from the drop in intensity for the primary scattering peak (color symbols) and enabled calculation of *χ*_PSMA‐PBzMA_=38.2/*T*−0.0393.

Characteristic domain spacings for these microphase‐separated materials could be calculated using the relation *d*=2π/q*, where q* is the position of the principal scattering vector. For lamellar phases, *d* corresponds to the thickness of two lamellar layers (also known as the “pitch” or *L*_0_), hence the individual domain spacing is described by the half‐pitch (*L*_0_/2). For cylindrical phases, *d* corresponds to the [100] inter‐planar distance between cylinders, and the mean cylinder width (*D*) is calculated from $D=d\frac{2\sqrt{3}v}{\pi^{1/2}}$ , where *v* is the volume fraction of the minority block. All PSMA_11_–PTFEMA_y_ diblock copolymers displayed features smaller than 10 nm: a lamellar half‐pitch as narrow as 5.5 nm was observed for PSMA_11_–PTFEMA_49_, and cylinders as thin as 7.6 nm were obtained for PSMA_11_–PTFEMA_28_, see Table S1 for a summary of these calculated domain spacings.

SAXS analysis of these five PSMA_11_–PTFEMA_y_ diblock copolymers confirmed that strong segregation in the bulk correlates with their self‐assembly to form PSMA‐stabilized nanoparticles with TFEMA cores in *n*‐tetradecane during PISA (Figure 1 b). SAXS patterns for higher molecular weight copolymers (*y*=28, 39 or 49) could be satisfactorily fitted using a spherical micelle model developed by Pedersen et al.,^16^ with mean core diameters ranging from 5.2 to 7.1 nm (see Figure S4 and Table S2 for further details of this model). In contrast, the weak minima observed for *y*=9 and *y*=18 suggests the formation of rather ill‐defined *pre‐micellar* aggregates coexisting with dissolved chains, which is consistent with prior studies of this PISA formulation.^13^ The broad Bragg peaks observed for these two copolymers in the bulk indicate the presence of disordered phases and/or weak segregation. This suggests that PISA could be used as a convenient screening tool. Micellar nucleation during PISA occurs despite the presence of unreacted monomer, which acts as a co‐solvent for the growing chains. Thus, targeting the *instantaneous* diblock copolymer composition corresponding to nucleation for a *final* diblock copolymer (i.e., that contains little or no residual monomer) should ensure that microphase separation definitely occurs in the bulk. Micellization can therefore be used to identify the minimum degree of polymerization that is required for the structure‐directing block to produce long‐range order in the bulk and minimal D‐spacings. In summary, these five PSMA_11_–PTFEMA_y_ diblock copolymers provide convenient access to a wide range of morphologies with sub‐10 nm domains by systematically varying just one parameter—the mean degree of polymerization of the PTFEMA block.

In addition to screening bulk phase behavior, PISA can be employed as a convenient method for rapid diblock copolymer syntheses that enable calculation of the Flory–Huggins interaction parameter. For example, a series of poly(stearyl methacrylate)–poly(benzyl methacrylate) (PSMA–PBzMA, see Scheme 1 c) diblock copolymers with approximately equal block volume fractions were prepared via PISA in mineral oil (Table S1). After isolation via precipitation, each copolymer underwent microphase separation in the bulk to produce a lamellar phase (Figure 1 c). SAXS was used to determine the order‐disorder transition temperature (*T*_ODT_) and hence establish a temperature‐dependent *χ* relationship such that *χ*_PSMA‐PBzMA_=38.2/*T*−0.0393 (see Figure 1 d and the Experimental section for further details). The combination of PISA synthesis and SAXS analysis therefore facilitates the identification of strongly‐segregated pairs of polymers that undergo self‐assembly to form sub‐10 nm domains.

**Casting diblock copolymer films from nanoparticle dispersions prepared via PISA**. In principle, aqueous PISA offers an efficient, environmentally‐friendly route to well‐defined nanoparticles at up to 50 % w/w solids.10a, 10d, 17 We hypothesized that waterborne diblock copolymer dispersions prepared via PISA could be *directly* employed to produce thin films for applications including nanolithography.3b Thus, sterically‐stabilized poly(glycerol monomethacrylate)–poly(2‐(diisopropylamino)ethyl methacrylate) (PGMA_28_–PDPA_21_; Scheme 1 d) nanoparticles were synthesized via RAFT aqueous emulsion polymerization of DPA at 20 % w/w solids. Thin films prepared using the as‐synthesized PISA dispersion were compared to that of the same diblock copolymer molecularly dissolved in a 1:1 w/w chloroform/methanol mixture. In the former case the amphiphilic copolymer chains are pre‐organized as self‐assembled nanoparticles (or copolymer micelles) during PISA, whereas in the latter case this initial order is lost during nanoparticle dissolution.

SAXS analysis confirmed that (i) the aqueous dispersion of PGMA_28_–PDPA_21_ nanoparticles comprised well‐defined spheres with a volume‐average core diameter of approximately 10.3 nm and (ii) molecular dissolution of this diblock copolymer occurs in the 1:1 w/w chloroform/methanol mixture (Figure S5). Films prepared by spin‐coating a 20 % w/w nanoparticle dispersion or copolymer solution onto mica were analyzed by transmission SAXS analysis (Figure S6). The much narrower scattering peaks in the former case suggested that significantly greater order is achieved (the full‐width half‐maximum, FWHM, is only 0.0082 after annealing at 100 °C for the nanoparticle‐cast films compared to 0.0140 for the solution‐cast film).

Grazing‐incidence small‐angle X‐ray scattering (GISAXS) was employed to assess the early stage development of domain alignment at the copolymer‐air interface upon annealing. Azimuthal angle vs. intensity plots recorded during a temperature step ramp show that domains in nanoparticle‐cast copolymer films become increasingly *aligned perpendicular to the interface with air* upon heating to 140 °C (Figures 2 a and b), as indicated by the appearance of scattering cones (Figure 2 a inset). In contrast, solution‐cast films exhibit more isotropic orientation with stronger preference for *parallel alignment to the interface*, as indicated by the horizontal diffuse green band observed in the early stages of annealing up to 140 °C (Figure 2 b inset).

**Figure 2:**
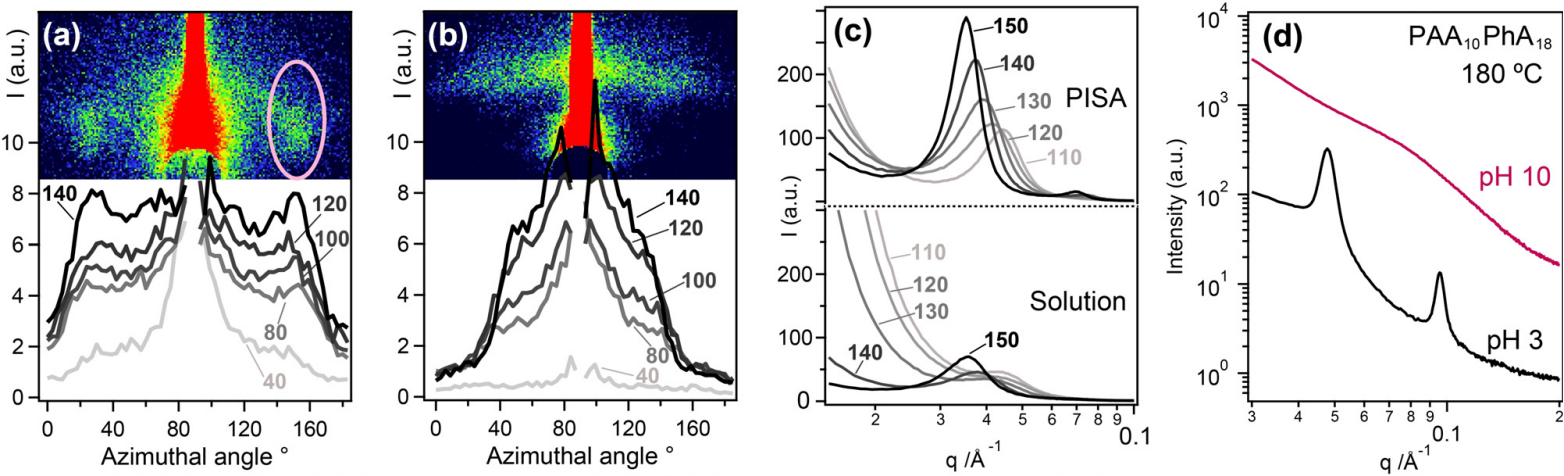
Diblock copolymer nanoparticles offer decisive processing advantages over molecularly‐dissolved diblock copolymer chains. Azimuthal GISAXS profiles recorded on heating from 40 to 140 °C and 2D GISAXS images obtained at 140 °C (inset) for copolymer films cast from a) a 10 % w/w aqueous dispersion of PGMA_28_–PDPA_21_ nanoparticles and b) the same copolymer after its molecular dissolution in a 1:1 w/w chloroform/methanol mixture. c) Solid‐state SAXS patterns obtained on heating bulk samples of PGMA_28_–PDPA_21_ from 110 to 150 °C. PGMA_28_–PDPA_21_ was isolated by either drying nanoparticles at pH 6.8 (top) or drying molecularly dissolved chains in 0.01 m HCl (bottom). d) Solid‐state SAXS data for PAA_10_‐PPhA_18_ freeze‐dried from aqueous solution at pH 10 (red curve) and pH 3 (black curve).

The enrichment of perpendicular structures close to the film/air interface was confirmed by AFM analysis, with such features persisting after annealing for 12 h at 140 °C (Figure S7a and S7b). Such domain alignment at the interface is desirable in applications where patterning of the underlying substrate is desired.^1^ Importantly, the surface roughness obtained for films prepared from nanoparticle dispersions was no greater than that observed for the equivalent solvent‐cast film after annealing, indicating that using nanoparticles does not lead to any additional surface roughness. However, we recognize that the high level of defects in these films would require further process optimization (e.g. substrate treatments, annealing environments^18^) to produce sufficiently well‐ordered structures for lithography applications. Nevertheless, these data demonstrate that a copolymer dispersion prepared via PISA can provide more convenient access to phase‐separated microstructures than the equivalent molecularly‐dissolved copolymer solution.

For aqueous dispersions of pH‐responsive amphiphilic diblock copolymers, the solution pH can be used to control the copolymer morphology.^19^ Protonation of the tertiary amine groups on the hydrophobic core‐forming PDPA block within PGMA_28_‐PDPA_21_ nanoparticles occurs on lowering the solution pH, resulting in molecular dissolution of the copolymer chains. Isolation of PGMA_28_‐PDPA_21_ nanoparticles by drying at pH 6.8 (the PDPA block is less than 50 % protonated under such conditions19a) produced a well‐ordered lamellar phase with *L*_0_=17.8 nm after slowly heating from 110 to 150 °C (Figure 2 c, top), as evidenced by equally‐spaced sharp Bragg peaks. In contrast, SAXS analysis of fully‐protonated PGMA_28_‐PDPA_21_ copolymer chains dried from a 10 mm HCl solution and annealed under the same conditions revealed a single broad low‐intensity peak. This feature indicates substantial disorder and did not change significantly when heating from 110 to 150 °C, (Figure 2 c, bottom).

Similarly, the degree of ionization of the steric stabilizer block in poly(acrylic acid)–poly(phenyl acrylate) (PAA_10_–PPhA_18_) nanoparticles prepared by RAFT aqueous emulsion polymerization10g can be controlled by varying the solution pH (see Scheme 1). However, in this case the original spherical morphology is retained and no molecular dissolution occurs. Isolation of PAA_10_–PPhA_18_ nanoparticles from mildly acidic solution (pH 3) produced a well‐ordered lamellar phase with *L*_0_=13.2 nm after annealing at 180 °C, as indicated by a series of sharp Bragg peaks (Figure 2 d, bottom). However, the same nanoparticles isolated in their fully ionized, highly anionic form by freeze‐drying a mildly alkaline solution (pH 10) exhibited only broad, ill‐defined features when analyzed by SAXS at 180 °C (Figure 2 d, top). Moreover, only a disordered solid‐state structure was obtained after extensive annealing above 250 °C, which is the literature *T*_g_ value for poly(sodium acrylate).^20^ The highly anionic coronal chains are mutually repulsive under such conditions, which prevents nanoparticle coalescence to form periodic long‐range structures in the solid state. At low pH, hydrogen‐bonded carboxylic acid dimers^21^ can be formed that favor coalescence and the formation of structures with long‐range order.

Therefore, tuning the solution pH can be a powerful and convenient tool to control block copolymer morphologies in the solid state. The pre‐organization of diblock copolymer chains within the sterically‐stabilized nanoparticles afforded by PISA prior to thin film fabrication clearly facilitates microphase separation. Similar observations were recently reported for poly(1,1‐dimethyl silacyclobutane)–poly(methyl methacrylate) diblock copolymer films cast from nanoparticle dispersions prepared via traditional post‐polymerization processing.^22^ However, PISA offers decisive advantages over post‐polymerization processing for industrial scale‐up, since the latter approach invariably involves multiple steps, toxic organic co‐solvents and relatively dilute copolymer solutions. Overall, aqueous PISA formulations enable both the efficient synthesis and convenient processing of high *χ*–low *N* diblock copolymers, allowing the facile preparation of thin films with small feature sizes (in this case with *L*_0_/2=8.9 nm).

**PISA synthesis of etchable diblock copolymers**. Block copolymer materials with selective etchability are invaluable for the preparation of patterned surfaces or porous materials for many potential applications.3a Polydimethylsiloxane (PDMS) and other silicon‐containing polymers are highly desirable for nanolithography‐based applications on account of their excellent resistance to oxygen plasma compared to conventional organic polymers.^23^ Monocarbinol‐terminated PDMS can be readily transformed via esterification to produce a macromolecular chain transfer agent suitable for RAFT polymerization: such polymers have been recently reported to be effective steric stabilizers for a wide range of core‐forming methacrylic blocks via PISA syntheses conducted in non‐polar solvents such as *n*‐heptane or silicone oil.^24^ In order to maximize both the *χ* parameter and the etch selectivity between the two blocks, we selected 2‐hydroxypropyl methacrylate (HPMA) as the insoluble core‐forming block. PHPMA is a relatively polar polymer that is known to be more susceptible to oxygen plasma than common vinyl polymers such as polystyrene or poly(methyl methacrylate).^25^ Accordingly, PDMS–PHPMA diblock copolymer nanoparticles (Scheme 1 f) were prepared via PISA in *n*‐heptane. After drying under vacuum and annealing at 120 °C, PDMS_16_–PHPMA_12_ underwent microphase separation in the bulk to form well‐ordered lamellae with *L*_0_=10.7 nm (Figure 3 a). In contrast, the absence of any well‐defined higher order peaks in PDMS_66_–PHPMA_30_ precluded reliable phase assignment by SAXS.

**Figure 3:**
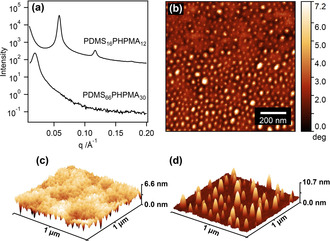
Etchable high–*χ* low‐*N* diblock copolymers prepared via PISA. a) SAXS patterns recorded for two PDMS_x_–PHPMA_y_ copolymers in the solid state immediately after heating to 120 °C (PDMS_11_–PHPMA_8_) or 140 °C (PDMS_66_–PHPMA_30_) from 20 °C at 1 °C min^−1^. b) AFM phase image and c) AFM height image of a thin film prepared by solvent‐casting PDMS_66_–PHPMA_30_ nanoparticles from *n*‐heptane after synthesis by PISA. d) AFM height image showing the patterned surface obtained after mild etching of this PDMS_66_–PHPMA_30_ film under oxygen plasma for 10 seconds at 25 °C.

Films cast from 10 % w/w dispersions of PDMS–PHPMA nanoparticles in *n*‐heptane were annealed at 140 °C for 30 min prior to atomic force microscopy (AFM) studies. Phase images of PDMS_66_–PHPMA_30_ (Figure 3 b and Figure S9a) revealed structures with disordered hexagonal packing arranged perpendicular to the copolymer‐air interface, while height images indicated some surface roughness (Figure 3 c, RMS roughness=0.981 nm). GISAXS analysis of PDMS_66_–PHPMA_30_ after extended annealing revealed some broad higher order peaks, suggesting that the morphology was disordered hexagonally‐packed cylinders (Figure S8). Meanwhile, despite the superior long‐range order in the bulk, phase images obtained for PDMS_16_–PHPMA_12_ films lacked structure (Figure S9b). Presumably, this is because the PDMS block was enriched at the interface in this copolymer. Therefore, for the purposes of this study we selected PDMS_66_–PHPMA_30_ films with perpendicular cylinders for etching experiments.

After exposure to oxygen plasma for 10 s, a surface array of cylinders was obtained with a mean cross‐sectional diameter of 29.2±8.6 nm (Figure 3 d). The domains appear to be larger than the parent template, indicating that some aggregation occurred during the etching protocol. The surface roughness of the template may not be sufficiently low for lithography but further optimization is beyond the scope of the current study. Nevertheless, it is clear that nanopatterned surfaces can be obtained by selective etching of a diblock copolymer film generated by drying nanoparticles, rather than molecularly‐dissolved copolymer chains.

In summary, we demonstrate that RAFT‐mediated PISA is an attractive and convenient route to high *χ*–low *N* diblock copolymers that form well‐ordered nanostructured materials in the solid state. In the case of PSMA_11_–PTFEMA_*y*_ nanoparticles, a range of copolymer morphologies can be accessed by adjusting the mean degree of polymerization of the core‐forming PTFEMA block, and the minimum DP required to ensure well‐defined solid‐state morphologies correlated well with that required for the onset of micellization in solution. Pre‐organization of the diblock copolymer chains in PGMA_28_–PDPA_21_ nanoparticles significantly expedites the onset of ordering during thin film and bulk material preparation compared to the corresponding molecularly‐dissolved copolymer chains under the same processing conditions. In principle, this should facilitate lower annealing temperatures and/or shorter annealing times. Solvents commonly employed in PISA, for example water, lower alcohols or *n*‐alkanes, also offer environmentally‐friendly alternatives compared to the conventional organic solvents typically employed for solution casting. Finally, an example of a new etchable PDMS‐based diblock copolymer is reported that can be readily prepared via PISA. The efficiency, convenience and potential scalability of this powerful platform technology offers decisive advantages over the complex multi‐step synthesis and processing steps that are all too often required to produce conventional high *χ*–low *N* diblock copolymers.

## Conflict of interest

The authors declare no conflict of interest.

## Supporting information

As a service to our authors and readers, this journal provides supporting information supplied by the authors. Such materials are peer reviewed and may be re‐organized for online delivery, but are not copy‐edited or typeset. Technical support issues arising from supporting information (other than missing files) should be addressed to the authors.

Supplementary
