# Supplementary material for: Synthesis of High χ–Low N Diblock Copolymers by Polymerization‐Induced Self‐Assembly
Source: Angew Chem Int Ed Engl. 2020 May 4;59(27):10848–53. doi: 10.1002/anie.202001436 (PMC7317809; doi:10.1002/anie.202001436)
Supplement: Supplementary file 1 — Supplementary [file ANIE-59-10848-s001.pdf]

Angewandte Chemie
Eine Zeitschrift der Gesellschaft Deutscher Chemiker

Supporting Information

Synthesis of High χ–Low N Diblock Copolymers by Polymerization-Induced Self-Assembly

James Jennings, Erik J. Cornel, Matthew J. Derry, Deborah L. Beattie, Matthew J. Rymaruk, Oliver J. Deane, Anthony J. Ryan, and Steven P. Armes**

anie_202001436_sm_miscellaneous_information.pdf

Summary of Supporting Information

Contents **Page**

Table S1: Structural characteristics of all the diblock copolymers examined in this study S2

Figure S1: GPC analysis for a series of five PSMA$_{11}$-PTFEMA$_{y}$ diblock copolymers S3

Figure S2: Temperature-dependent SAXS analysis for PSMA$_{11}$-PTFEMA$_{39}$ S4

Figure S3: Peak assignment for the gyroid phase in PSMA$_{11}$-PTFEMA$_{39}$ at 166 °C S5

Figure S4: Results of fitting spherical micelle models to PSMA$_{11}$-PTFEMA$_{y}$ SAXS patterns S6

Table S2: Variable parameters used to fit spherical micelle models to PSMA$_{11}$-PTFEMA$_{y}$ S6

Table S3: Constant parameters used to fit spherical micelle models to PSMA$_{11}$-PTFEMA$_{y}$ S7

Table S4: Results from the spherical micelle model fits for PSMA$_{11}$-PTFEMA$_{y}$ S7

Figure S5: SAXS analysis of precursors used for solvent casting of films S7

Figure S6: Transmission SAXS analysis of films cast from PGMA$_{28}$-PDPA$_{21}$ S8

Figure S7: AFM phase images of PGMA$_{28}$-PDPA$_{21}$ prepared under different conditions S8

Figure S8: Integrated GISAXS of PDMS$_{66}$-PHPMA$_{30}$ with peak fitting for cylindrical phase S9

Figure S9: Additional AFM phase images for PDMS-PHPMA copolymer films S9

Experimental Details S10

References S16

Table S1. Structural characteristics of the various diblock copolymers studied in this paper, including the mean degrees of polymerization (DP) of each block, calculated volume fractions (φ_1), number-average molecular weight (M_n) and dispersity (M_w/M_n).

Stabilizer Block (DP)	Core Block (DP)	Solvent	φ_1[a]	GPC		Bulk Morphology	Characteristic dimension[b]/nm
				M_n	M_w/M_n		
PSMA (11)	PTFEMA (9)	*n*-tetradecane	0.806	7.1	1.11	Disorder	-
PSMA (11)	PTFEMA (18)	*n*-tetradecane	0.676	8.5	1.11	Disorder	-
PSMA (11)	PTFEMA (28)	*n*-tetradecane	0.573	9.4	1.14	C	**D = 7.6**
PSMA (11)	PTFEMA (39)	*n*-tetradecane	0.490	10.9	1.16	C/G	**D = 9.8**
PSMA (11)	PTFEMA (49)	*n*-tetradecane	0.434	12.1	1.15	L	**L_0 = 12.6**
PSMA (31)	PBzMA (76)	mineral oil	0.500	19.9	1.20	L	**L_0 = 18.9**
PSMA (18)	PBzMA (54)	mineral oil	0.450	14.3	1.13	L	**L_0 = 13.4**
PSMA (13)	PBzMA (31)	mineral oil	0.507	10.4	1.11	L	**L_0 = 10.3**
PAA (10)	PPhA (18)	water	0.235	4.7	2.00	L	**L_0 = 13.2**
PGMA (28)	PDPA (21)	water	0.409	7.4	1.34	L	**L_0 = 17.8**
PDMS (16)	PHPMA (12)	*n*-heptane	0.465	4.4	1.41	L	**L_0 = 10.7**
PDMS (66)	PHPMA (30)	*n*-heptane	0.582	19.2	1.15	C[c]	**D = 17.9**

[a] Volume fraction of stabilizer block calculated using ^{1}H NMR analysis in combination with literature data for melt densities.

[b] Calculated from SAXS data (where D refers to the cylinder width and L_0 to the lamellar full-pitch).

[c]Determined by analysis of AFM data and peak fitting of GISAXS patterns. In all other cases phase assignment was based on SAXS peak fitting.

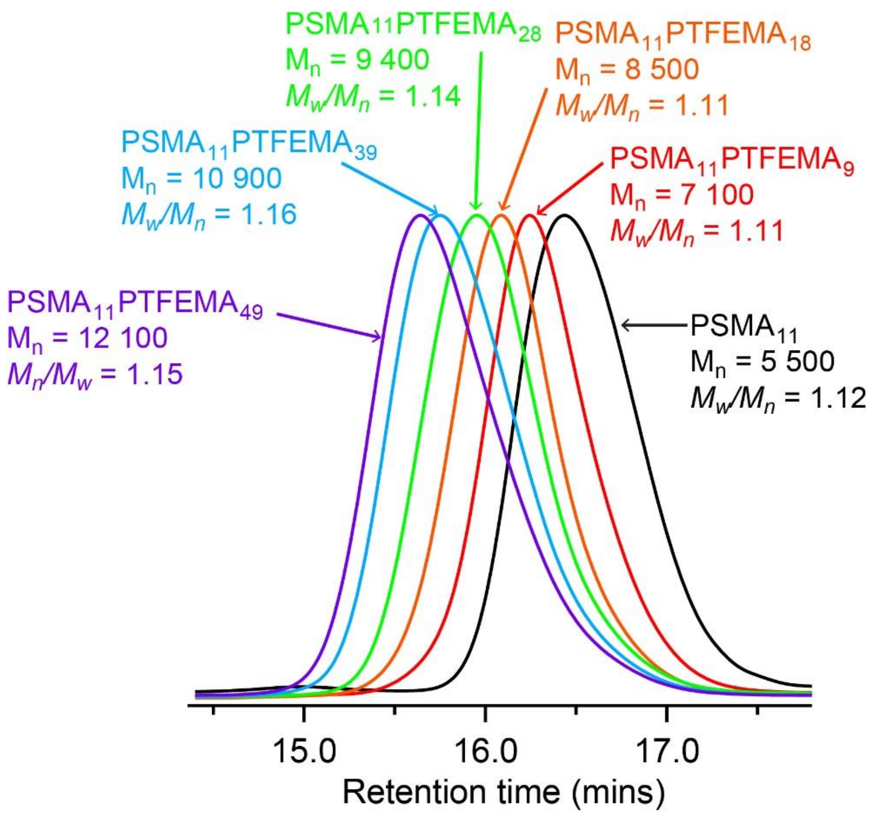

Figure S1. Normalized GPC curves (refractive index detector, THF eluent) recorded for $PSMA_{11}$ macro-CTA (black) and the series of five $PSMA_{11}$-$PTFEMA_x$ diblock copolymers investigated in this study.

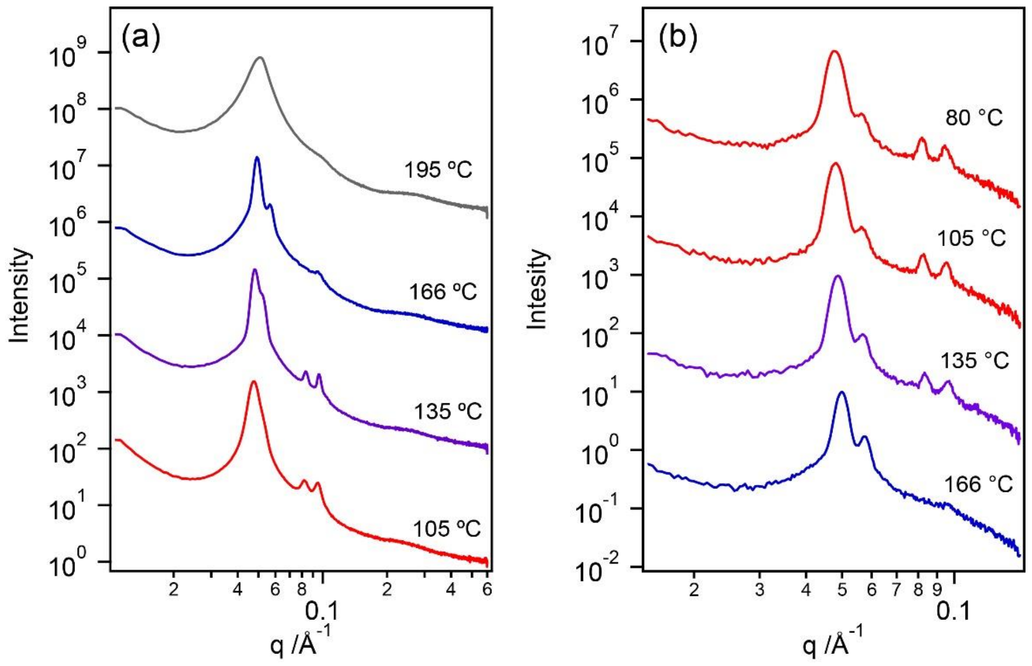

Figure S2. Temperature-dependent SAXS data recorded for PSMA$_{11}$-PTFEMA$_{39}$ upon heating (a) and cooling (b). Upon heating the cylindrical phase is observed at 105 °C (red), which transforms into a cylinder-gyroid mixed phase at 135 °C (purple) and a pure gyroid phase (apparent within the limits of instrument resolution) at 166 °C (blue) before undergoing an order-disorder transition (ODT) at 195 °C (grey). Upon cooling from the pure gyroid phase at 166 °C, the cylinder-gyroid mixed phase reappears. The lower resolution and noisier data in (b) is due to the different SAXS instrument used.

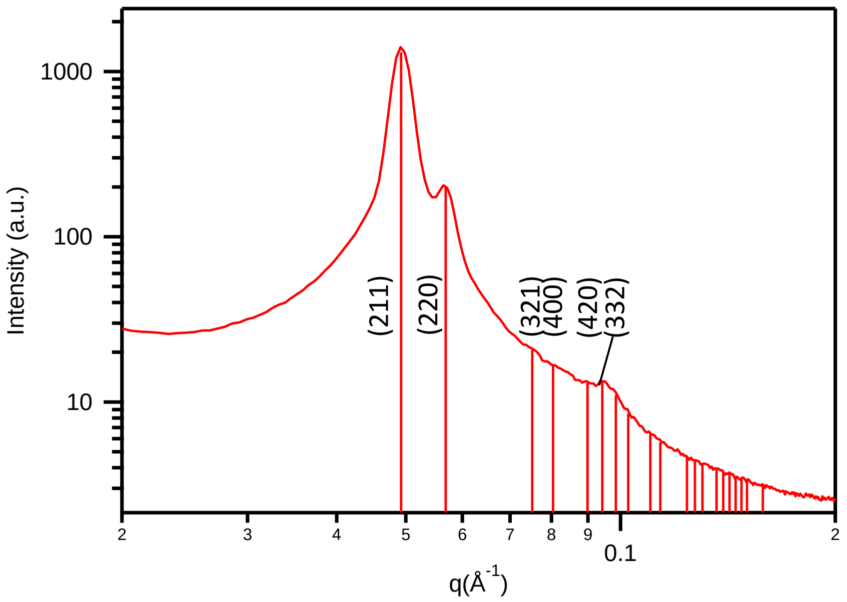

Figure S3. Peak assignment performed on SAXS data obtained for $PSMA_{11}$-$PTFEMA_{39}$ at 166 °C using a line-fitting macro for the gyroid phase in Igor Pro®[1]. Lines represent the expected peak positions arising from the gyroid morphology and are labelled with the six Miller indices that were resolved in this pattern.

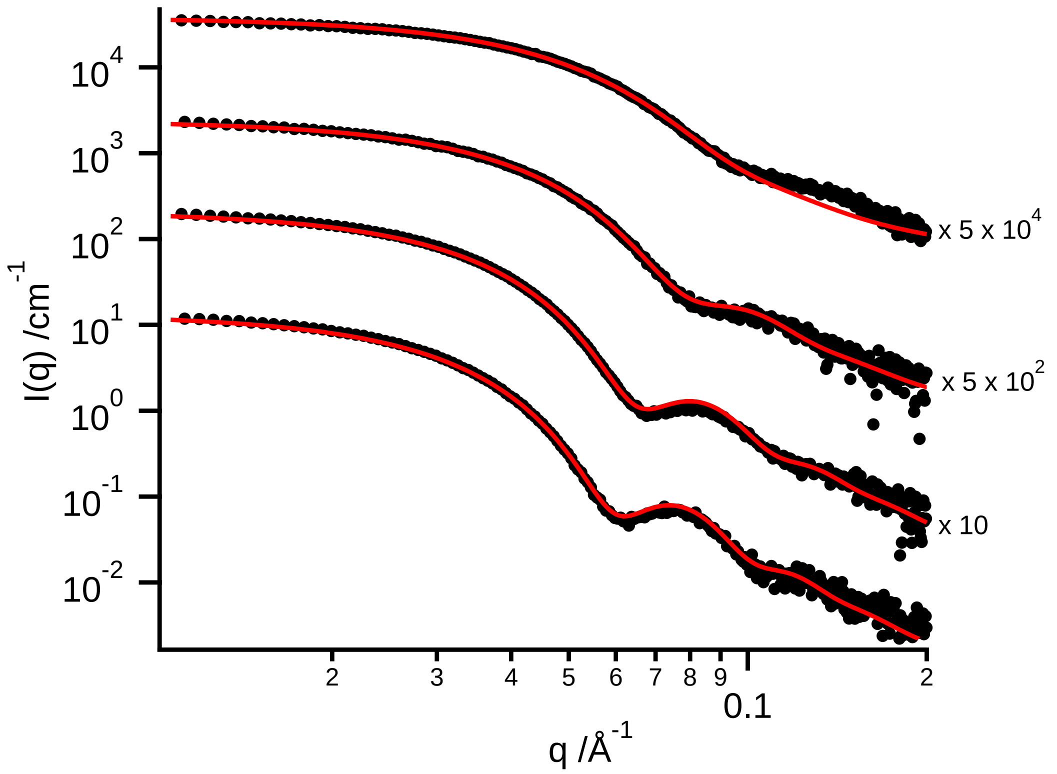

Figure S4. SAXS patterns recorded for 1.0% w/w dispersions of $PSMA_{11}$−$PTFEMA_{x}$ in *n*-tetradecane (black circles) overlaid with the red fitting curves obtained from a spherical micelle model (Gaussian size distribution) developed by Pedersen *et al*[2] using Igor Pro®. The variables and constants used in the fitting are listed in Table S2 and S3, respectively, and results from the fittings listed in Table S4. From top to bottom, data fits yielded core radii of 4.2, 5.2, 6.5 and 7.1 nm for $PSMA_{11}$−$PTFEMA_{18}$, $PSMA_{11}$−$PTFEMA_{28}$, $PSMA_{11}$−$PTFEMA_{39}$, $PSMA_{11}$−$PTFEMA_{49}$, respectively. The unusually small value for shell R_g, larger standard deviation and lower scale (i.e. concentration) from the model for $PSMA_{11}$−$PTFEMA_{18}$ indicates ill-defined aggregates coexisting with dissolved chains. This is not unexpected, as this target diblock composition is barely sufficient to induce micellization during PISA.[3]

Table S2: Variable parameters used in the spherical micelle model to fit the SAXS data shown in Figure S4.

Polymer	PTFEMA volume / Å^3	Shell R_g /nm	Scale
$PSMA_{11}$−$PTFEMA_{18}$	3418	1.02	0.0005
$PSMA_{11}$−$PTFEMA_{28}$	5317	2.34	0.0008
$PSMA_{11}$−$PTFEMA_{39}$	7406	2.93	0.0008
$PSMA_{11}$−$PTFEMA_{49}$	9305	2.87	0.0008

Table S3: Fixed parameters used in the spherical micelle model to fit the SAXS data shown in Figure S4.

Polymer	PSMA volume / $Å^3$	SLD_{PSMA} ($x10^{-6}$)/ $Å^{-2}$	SLD_{PTFEMA} ($x10^{-6}$)/ $Å^{-2}$	Volume fraction solvent in core	Sigmoidal interface term /nm
$PSMA_{11}$−$PTFEMA_y$	6948	9.237	12.76	0	0.22

Table S4: Physical results of the spherical micelle models used to fit the SAXS data shown in Figure S4.

Polymer	Core radius /nm	Standard deviation /nm	N_{agg}	Volume fraction solvent in shell
$PSMA_{11}$−$PTFEMA_{18}$	4.2	1.0	91	0.86
$PSMA_{11}$−$PTFEMA_{28}$	5.2	0.8	110	0.84
$PSMA_{11}$−$PTFEMA_{39}$	6.5	0.8	155	0.78
$PSMA_{11}$−$PTFEMA_{49}$	7.1	0.8	161	0.11

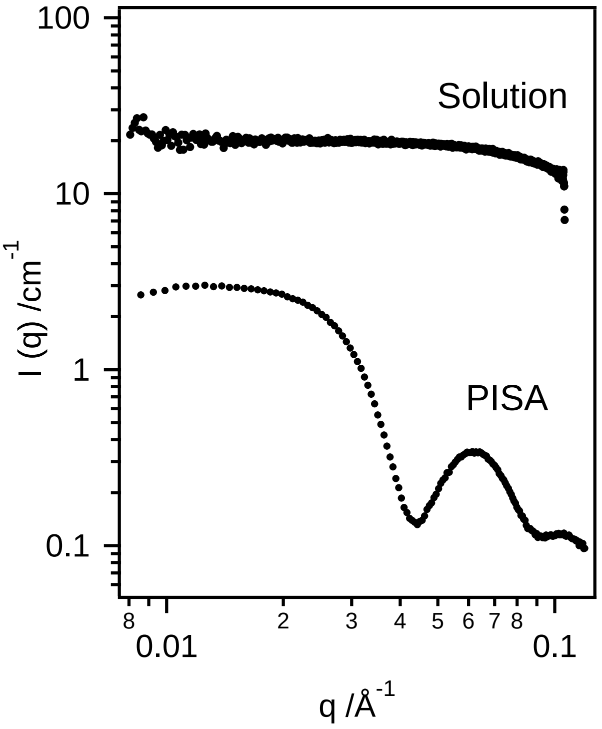

Figure S5: SAXS patterns recorded for a 20% w/w aqueous dispersion of $PGMA_{28}$-$PDPA_{21}$ nanoparticles prepared via PISA and the same diblock copolymer dissolved as individual chains in a 1:1 w:w chloroform/methanol mixture.

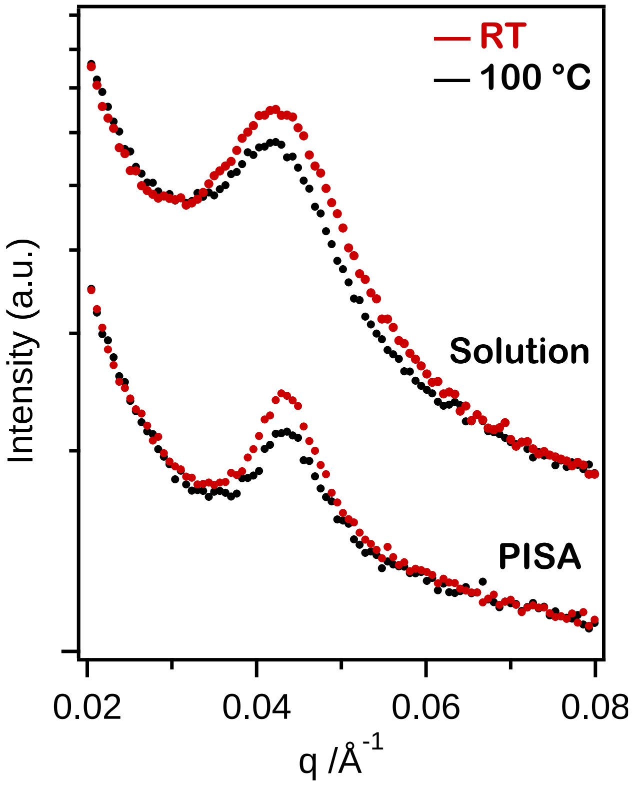

Figure S6. Transmission SAXS analysis of films prepared by spin-coating either PISA-synthesized nanoparticles or the corresponding diblock copolymer dissolved in 1:1 chloroform/methanol (w:w) onto mica disks. SAXS data were recorded on the as-spun films at 20 °C and also after heating to 100 °C under vacuum.

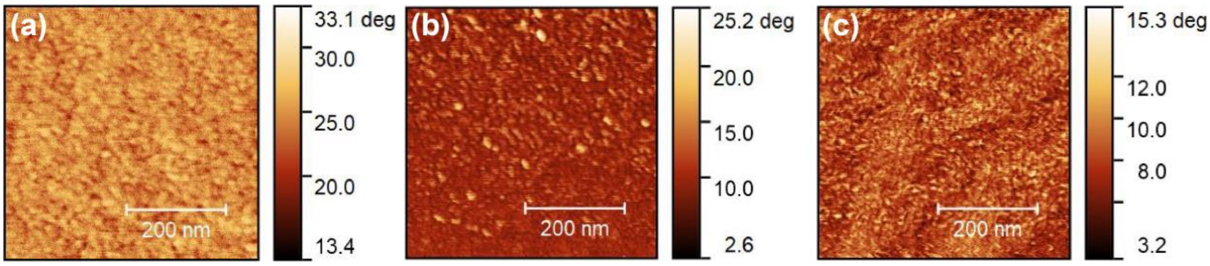

Figure S7. AFM images of PGMA$_{28}$-PDPA$_{21}$ prepared by (a) spin casting from PISA dispersion followed by annealing at 140 °C for 5 minutes, (b) spin casting from PISA dispersion followed by annealing at 140 °C for 12 h, and (c) spin casting from chloroform/methanol (1:1 w:w) followed by annealing at 140 °C for 12 h.

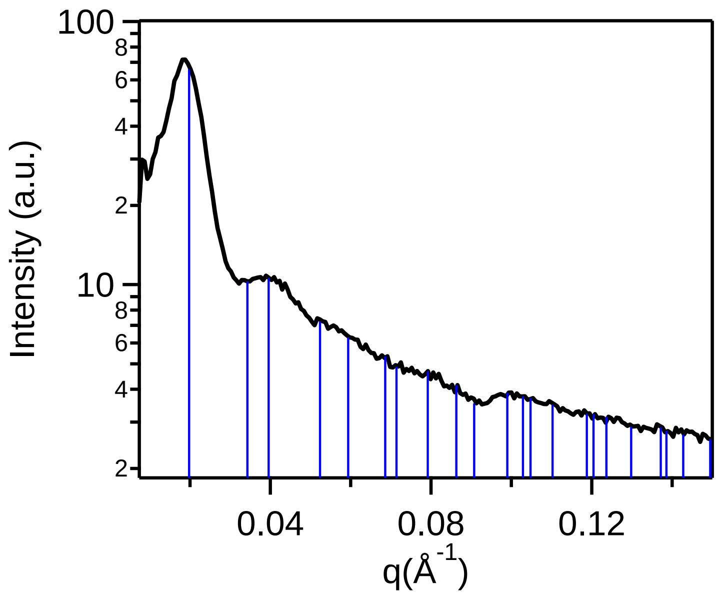

Figure S8: 1D trace obtained by integration of GISAXS data collected from a $PDMS_{66}PHPMA_{30}$ film after annealing overnight at 120 °C. The series of lines represent expected peak positions for the cylindrical phase, from a line-fitting macro in Igor Pro®[1]. These data corroborate that the film comprises a relatively disordered cylindrical morphology.

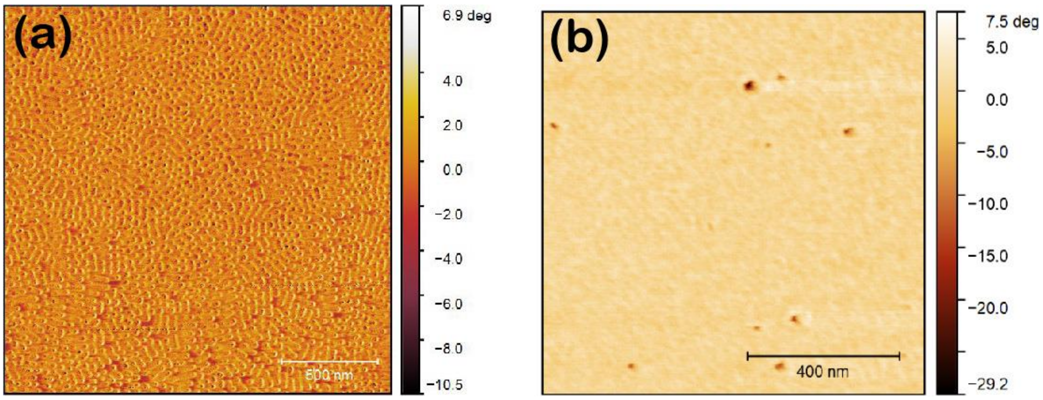

Figure S9. (a) AFM phase image of $PDMS_{66}PHPMA_{30}$ produced from spin coating, showing a higher proportion of parallel cylinders. (b) AFM phase image of $PDMS_{16}PHPMA_{12}$ film produced from spin coating showing a featureless surface, most likely due to enrichment of a PDMS lamellar layer at the interface.

Experimental Details

Materials. Stearyl methacrylate (SMA), 2,2,2-trifluoroethyl methacrylate (TFEMA), *n*-dodecane, *n*-tetradecane, *n*-hexane, trimethylamine, butylhydroxytoluene (BHT) tetrahydrofuran (THF), toluene, 2,2-azobis(2-methylpropionitrile) (AIBN), lauroyl peroxide (Luperox®), cumyl dithiobenzoate (CDB), benzyl methacrylate (BzMA), sodium periodate, deuterium oxide (D_2O) and deuterium chloride (DCl; 35% w/w in D_2O) were all purchased from Sigma-Aldrich (UK). CD_2Cl_2 was purchased from Goss Scientific (UK). 2,2'-Azobis[2-(2-imidazolin-2-yl)propane]dihydrochloride (VA-044) was obtained from Fluorochem (Hadfield, UK). 2-(Diisopropylamino)ethyl methacrylate (DPA) was purchased from Scientific Polymer Products Inc. (Ontario, NY). Hydrochloric acid (35 %) and $CDCl_3$ were obtained from VWR Chemicals UK. A 4 cSt American Petroleum Institute (API) group III mineral oil was kindly provided by The Lubrizol Corporation Ltd. (Hazelwood, Derbyshire, UK). Tert-Butyl peroxy-2-ethylhexanoate (T21s) initiator was purchased from AkzoNobel (The Netherlands). CD_2Cl_2 and d_8-toluene were obtained from Cambridge Isotope Laboratory (USA). Monomers were passed through basic alumina prior to use to remove inhibitor. The RAFT agent 4-cyano-4-((2-phenylethanesulfonyl)thiocarbonylsulfanyl)pentanoic acid (PETTC) was prepared in-house according to a previously reported protocol[4] and all materials were used as received unless otherwise noted. Mica disks (0.025 x 15.0 mm) were purchased from Attwater & Sons Ltd (Preston, UK). Borosilicate capillaries were purchased from WJM-Glass Muller GMBH (Berlin, Germany).

Synthesis of PSMA macro-RAFT agents. For example, $PSMA_{11}$ macro-CTA was prepared via RAFT solution polymerization of SMA in toluene. Briefly, SMA (19.95 g, 58.91 mmol), PETTC (2.00 g, 5.89 mmol; target DP = 10), AIBN (0.19 g, 1.18 mmol; PETTC/AIBN molar ratio = 5.0) and toluene (22.14 g) were added to a 100 ml round-bottomed flask. This reaction solution was heated by immersing the flask in an oil bath set at 70 °C and the resulting SMA polymerization was quenched by exposure to air after 4 h. ^{1}H NMR analysis in $CDCl_3$ indicated that 72% SMA conversion was achieved under these conditions. The crude product was precipitated in excess ethanol and dried under vacuum at 30 °C. A mean DP of 11 was determined for the purified PSMA macro-CTA via ^{1}H NMR analysis in CD_2Cl_2; the integrated aromatic PETTC signals at 7.1–8.1 ppm were compared to that of the oxymethylene signal at 3.7–4.2 ppm. GPC analysis in THF indicated an M_n of 5 400 g mol^{-1} and an M_w/M_n of 1.15.

Synthesis of $PSMA_{11}$-$PTFEMA_X$ nanoparticles by RAFT dispersion polymerization in *n*-tetradecane. The synthesis of $PSMA_{12}$-$PTFEMA_{49}$ nanoparticles at 20% w/w solids was conducted as follows: a $PSMA_{11}$ macro-CTA (0.15 g, 0.034 mmol), lauroyl peroxide (5.40 mg, 0.014 mmol), and *n*-tetradecane (1.75 g, 2.29 ml) were added in turn to a glass vial and the

resulting solution was degassed with N_2 gas for 30 min at 20 °C. TFEMA was degassed separately at 20 °C to minimize any evaporation losses. TFEMA (0.24 ml, 1.70 mmol; target DP = 50) was then added via syringe to the reaction mixture, which was subsequently heated to 70 °C for 16 h by immersing the vial in an oil bath. ^{1}H NMR analysis indicated a mean degree of polymerization of 49 for the PTFEMA block. The resulting dispersion of nanoparticles was precipitated into ethanol to remove traces of unreacted TFEMA monomer.

Synthesis of PSMA-PBzMA nanoparticles by RAFT dispersion polymerization in mineral oil. The PSMA-PBzMA diblock copolymer nanoparticles were synthesized according to the literature.[5] A typical RAFT dispersion polymerization synthesis of $PSMA_{31}$-$PBzMA_{76}$ diblock copolymer nanoparticles at 20 % w/w solids was conducted as follows. Benzyl methacrylate (BzMA; 0.212 g; 1.20 mmol), T21s initiator (0.666 mg; 3.1 μmol; dissolved at 10.0 % v/v in mineral oil) and $PSMA_{31}$ macro-CTA (0.166 g; 15.4 μmol; macro-CTA/initiator molar ratio = 5.0; target degree of polymerization for the PBzMA block = 78) were dissolved in mineral oil (1.51 g). The reaction mixture was sealed in a 10 mL round-bottomed flask and purged with nitrogen gas for 30 min. The deoxygenated solution was then placed in a pre-heated oil bath at 90 °C for 5 h. The crude diblock copolymer nanoparticles were purified by precipitation into a ten-fold excess of ethanol (twice), followed by drying in a vacuum oven to constant weight.

Synthesis of $PGMA_{28}$-$PDPA_{25}$ nanoparticles via RAFT aqueous emulsion polymerization. $PGMA_{28}$ macro-CTA was prepared by solution polymerization in ethanol as previously described.[6] $PGMA_{28}$ macro-CTA (0.4098 g, 0.087 mmol), VA-044 initiator (5.6 mg, 0.017 mmol; $PGMA_{28}$ macro-CTA/VA-044 molar ratio = 5.0) and DPA monomer (0.3903 g, 1.8 mmol) were weighed into a 14 mL vial before adding water (2.6007 g), 2 M HCl (0.1250 g) and 1 M HCl (0.4990 g) to produce a 20 % w/w solution with an initial pH of 7.6. The resulting emulsion was purged with nitrogen gas for 45 min at 25 °C before immersing the reaction vial into an oil bath set to 44 °C. The reaction solution was stirred overnight (17 h) to ensure high DPA monomer conversion (more than 99 % according to ^{1}H NMR analysis) before quenching the polymerization by exposing the vial contents to air and cooling to 25 °C. The transparent, pink, free-flowing solution was then freeze-dried from water for analysis. DMF GPC analysis (refractive index detector) indicated an M_n of 7 400 g mol^{-1} and an M_w/M_n of 1.34.

Synthesis of PAA macro-RAFT agent. AA (10.00 g, 138.8 mmol), DDMAT RAFT agent (1.687 g, 4.626 mmol; target DP = 30), AIBN (0.082 g, 0.463 mmol; DDMAT/AIBN molar ratio = 10) and ethanol (7.845 g, 60% w/w), were weighed into a 50 mL round-bottomed flask charged with a magnetic flea. This flask was then placed in an ice bath and degassed with nitrogen for 30 min, before being immersed in an oil bath set at 70 °C. The polymerization

was allowed to proceed for 70 min before being terminated by exposing the reaction solution to air while immersing the flask in an ice bath. ^{1}H NMR analysis indicated a monomer conversion of 55%. The crude PAA homopolymer was purified by dialysis against deionized water over 72 h to afford a PAA macro-CTA with a mean DP of 10 and less than 1% residual monomer, as measured by ^{1}H NMR spectroscopy. Prior to GPC analysis, PAA_{10} was methylated by adding excess trimethylsilyldiazomethane dropwise to a solution of PAA_{10} (20 mg) in THF (2.0 mL) until a persistent yellow coloration was observed. This reaction solution was then stirred overnight until all the solvent and excess reagent had evaporated prior to GPC analysis. THF GPC analysis (refractive index detector) indicated an M_n of 3 700 g mol^{-1} and an M_w/M_n of 1.07.

Synthesis of PAA-PPhA nanoparticles by RAFT aqueous emulsion polymerization. PAA_{10} macro-CTA (0.150 g, 0.138 mmol; target DP = 20), KPS (3.1 mg, 0.012 mmol; PAA_{10} macro-CTA/KPS molar ratio = 10.0) and mildly acidic water (0.889 g, pH 3) were weighed into a 10 mL vial charged with a magnetic flea (reaction solution 1). This vial was immersed in an ice bath and the aqueous solution was degassed with nitrogen for 30 min. Deionized water (1.000 g, corresponding to a 20% w/w solution) and AsAc (2.00 mg, 0.012 mmol; PAA_{10} macro-CTA/AsAc molar ratio = 10.0) were weighed into a separate 7 mL vial (reaction solution 2) and degassed with nitrogen gas using an ice bath for 30 min. PhA (2 mL) was weighed into a 7 mL vial and the solution was degassed with nitrogen for 30 min. Reaction solution 1 was then immersed in an oil bath set at 30 °C, and then reaction solution 2, followed by PhA (0.32 ml, 2.30 mmol), were added in turn to solution 1 under a nitrogen atmosphere. The PhA polymerization was allowed to proceed for 18 h before being quenched by exposure to air while immersing the reaction vial in an ice bath. ^{1}H NMR studies indicated more than 99% conversion and a mean DP of 18. THF GPC analysis after methylation (refractive index detector) indicated an M_n of 4 700 g mol^{-1} and an M_w/M_n of 2.00.

Synthesis of PDMS-PHPMA nanoparticles by RAFT dispersion polymerization in *n*-heptane. A monohydroxy-functional $PDMS_{66}$ precursor was reacted with carboxylic acid-functionalized PETTC RAFT agent to yield a $PDMS_{66}$-TTC macro-CTA, according to a previously reported esterification protocol.[7] A representative RAFT dispersion polymerization of HPMA in *n*-heptane, targeting a final PHPMA DP of 33 at 10 % w/w concentration was then conducted as follows: A 10 ml glass vial was charged with $PDMS_{66}$-TTC macro-CTA (0.10 g, 18.8 µmol), *n*-heptane (1.71 g), HPMA monomer (89.2 mg, 0.62 mmol) and a magnetic follower. T21s initiator was added as a 10 % v/v stock solution in *n*-heptane (1.35 mg, 6.26 µmol) and the vial was sealed with a rubber septum. This reaction mixture was then purged with nitrogen for 20 min, sealed, and placed in a pre-heated oil bath set at 70 °C for 16 h. The resulting dispersion was obtained as a free-flowing fluid and ^{1}H NMR analysis indicated a

HPMA conversion of 99 %. THF GPC analysis (refractive index detector) indicated an M_n of 19 200 g mol^{-1} and an M_w/M_n of 1.15.

Gel permeation chromatography (GPC). Molecular weight distributions for PSMA-PTFEMA, PSMA-PBzMA, PAA-PPhA, and PDMS-PHPMA were assessed by GPC using THF eluent. The THF GPC set-up consisted of an Agilent 1260 Infinity series degasser and pump, two Agilent PLgel 5 μm Mixed C columns in series and a refractive index detector. The mobile phase contained 2.0% v/v trimethylamine and 0.05% w/w butylhydroxytoluene (BHT) and the flow rate was fixed at 1.0 ml min^{-1}. Samples were dissolved in THF containing 0.50% v/v toluene as a flow-rate marker prior to GPC analysis. A series of near monodisperse poly(methyl methacrylate) standards were used for calibration of the refractive index detector. PGMA-PDPA was analysed in DMF eluent containing 10 mM LiBr.

^{1}H NMR. Spectra were recorded in deuterated solvents using either a Bruker AV1-400 or a AV1-250 MHz spectrometer. End-group analysis using protons from the RAFT agent was performed to calculate the mean DP.

Small Angle X-ray Scattering (SAXS) analysis. Solid-state and solution SAXS studies were conducted using a Xeuss 2.0 (Xenocs) SAXS instrument equipped with a FOX 3D multilayered X-ray mirror, two sets of scatterless slits for collimation, a hybrid pixel area detector (Pilatus 1M, Dectris) and a liquid gallium MetalJet X-ray source (Excillum, λ = 1.34 Å). SAXS patterns were recorded at a sample-to-detector distance of approximately 1.20 m (calibrated using a silver behenate standard). 2D SAXS patterns were reduced to 1D plots by azimuthal integration within the Foxtrot software package. The solution of PGMA$_{28}$-PDPA$_{25}$ in 1:1 chloroform/methanol was run on I22 at Diamond Light Source (λ = 0.999 Å), with SAXS patterns recorded on a 2M Pilatus at a sample-to-detector distance of 9.75 m.

Solution SAXS studies were conducted at 25 °C using borosilicate capillaries at a concentration of 1.0% w/w (PSMA$_{12}$-PTFEMA$_y$) or 20% w/w (PGMA$_{28}$-PDPA$_{25}$). After removal of the reaction solvent, the solid diblock copolymers were loaded into borosilicate capillaries and mounted on a HFSX350-CAP temperature-controlled stage (Linkam Scientific, Tadworth, UK) for temperature-dependent SAXS experiments. Detailed protocols to isolate each diblock copolymer in the solid-state and *in situ* heating profiles are outlined below.

PSMA-PTFEMA: After precipitation into excess ethanol, copolymers were dried under vacuum at 30 °C overnight. Capillary-loaded powders were first heated to 80 °C, i.e. above the T_m of PSMA (ca. 35 °C) and the T_g of PTFEMA (ca. 69 °C), then heated at a rate of 1 °C min^{-1} up to 240 °C, or the temperature at which a clear order-disorder transition (ODT) was observed. Lower molecular weight samples were heated from 30 to 80 °C, during which only disordered

phases were observed at all temperatures. During these heating cycle, a frame was recorded every minute using an exposure of 58 s.

PSMA-PBzMA: After precipitation into excess ethanol, samples were annealed under vacuum at 200 °C for 30 min. Capillary-loaded powders were first cooled to 10 °C, then heated at a rate of 1 °C min^{-1} up to 250 °C (or up to the temperature at which an order-disorder transition was first observed). During this heating cycle, a frame was recorded every minute from a 58 s exposure.

PGMA-PDPA: Solid diblock copolymer was isolated from two different precursor solutions: (i) directly from the aqueous nanoparticle dispersion by freeze-drying the final reaction solution (at pH 6.8, 20 % w/w) and (ii) after redissolving the solid isolated from (i) in 0.01 M HCl and freeze-drying this 20 % w/w solution. Freeze-dried powders were then loaded into capillaries, mounted onto the Linkam capillary stage, and heated from 50 to 150 °C under vacuum at a rate of 1 °C min^{-1}. During this heating cycle, a frame was recorded every minute from a 58 s exposure.

PAA-PPhA: Prior to separation from aqueous solution, the solution pH was adjusted to either pH 3 or pH 10 using 1 M HCl or 1 M NaOH to afford either poly(acrylic acid)- or poly(sodium acrylate)- stabilized dispersions, respectively. Solid diblock copolymers were then isolated by freeze-drying the dispersions and loaded into capillaries, before being annealed under vacuum at 140 °C for 1.5 h to ensure complete removal of water. Samples were then loaded onto the Linkam capillary stage and heated to either 75, 100, 125, 150, 160, 170 or 180 °C, annealed for 10 min at each temperature, and frames were collected for 58 s. Samples isolated from aqueous solutions at pH 10 were further annealed at 250 °C for 30 min in an attempt to achieve an ordered phase.

PDMS-PHPMA: n-Heptane was removed from the nanoparticle dispersion under vacuum at 80 °C overnight, and the resulting dry copolymer powders were loaded into capillaries and mounted on the Linkam capillary stage within the SAXS beamline. $PDMS_{66}$-$HPMA_{30}$ and $PDMS_{16}$-$HPMA_{12}$ were heated under vacuum from 30 to 120 °C at a rate of 1 °C min^{-1}. During these heating cycles, a frame was recorded every minute from a 58 s exposure. The samples were then held at 120 °C, during which a further thirty frames were collected.

Preparation of copolymer films from nanoparticle dispersions. For transmission SAXS analysis of $PGMA_{28}$-$PDPA_{25}$ diblock copolymers, films were prepared by spin-casting from 20% w/w dispersions (as-synthesized via PISA) or from the freeze-dried copolymer dissolved in a 1:1 w/w mixture of chloroform and methanol. $PGMA_{28}$-$PDPA_{25}$ copolymer films for GISAXS analysis were prepared by casting from nanoparticle dispersions (diluted to 10% w/w solids

using deionized water) or VOC solutions at the equivalent copolymer concentration. Precise volumes of either dispersion or solution (25 µl) were cast onto a mica disk spinning at 1 000 rpm using an Ossila spin-coater. After 2 min, the spin-coater speed was increased to 4 500 rpm for 10 min to ensure complete removal of residual solvent. $PDMS_{66}$-$PHPMA_{30}$ films for AFM analysis were prepared by drop-casting 25 µl of an as-synthesized 10% w/w nanoparticle dispersion in *n*-heptane and allowing the solvent to evaporate at 20 °C for at least 72 h.

Temperature-dependent transmission SAXS analysis of diblock copolymer films. Coated mica disks were attached to the heating plate of a HFSX350-CAP temperature-controlled stage using Kapton tape, before being mounted into the beamline (as described above). Films were incrementally heated under vacuum to 40, 60, 80, 100, 120, and 140 °C at a heating rate of 10 °C min^{-1}. At each temperature, SAXS patterns were recorded from three exposures each of five min duration.

Grazing Incidence Small Angle X-ray Scattering (GISAXS) analysis. Coated mica disks were placed on the heating plate of a custom-built temperature-controlled Linkam stage mounted within the beamline. Samples were analyzed under vacuum at 25 °C and 40, 60, 80, 100, 120 and 140 °C. After 5 min annealing at each temperature, patterns were recorded using 60 s exposures at an incident angle of $\alpha_i = 0.1°$. To convert 2D images into 1D plots, azimuthal profiles (Ψ vs. intensity) were obtained after adding a threshold mask to remove direct beam scattering (i.e. Ψ ~ 90°). Integrations were performed in steps of 3° to produce 1D plots in the region of the primary scattering peak (q* ± 0.01 $Å^{-1}$).

Order-disorder transition (ODT) measurements for PSMA-PBzMA. After precipitation into ethanol, PSMA-PBzMA copolymers were loaded into 2mm borosilicate capillaries and annealed at 200 °C under vacuum for 30 min. Capillaries were then mounted onto a Linkam capillary heating stage within the Xenocs Xeuss 2.0 beamline, and heated from 25 °C at 1 °C min^{-1} while collecting a 58 s frame every minute until the ODT was observed. This temperature (T_{ODT}) was determined as that at which the intensity (I) of the primary scattering peak is significantly reduced (plots of 1/I vs. 1/T are plotted in Figure 2d), which was corroborated with the temperature at which the FWHM of the primary scattering peak significantly increased (data not shown). The χ value at the ODT for each copolymer was calculated from $\chi N' = 10.5$, where N' is the volume-normalized degree of polymerization (normalized to a reference volume of 100 $Å^3$). Thus, the temperature-dependent χ could be obtained from a plot of $1/T_{ODT}$ vs. χ ($\chi_{PSMA\text{-}PBzMA} = 38.2/T - 0.0393$).

Atomic Force Microscopy (AFM). AFM studies were performed using a Veeco Dimension 3100 instrument equipped with a Nanoscope 3A feedback controller. A TESPA-V2 probe with a z-limit of 4 µm was employed in tapping mode. 1 x 1 µm phase and height images

were recorded using 512 line scans. Etching was performed by exposing films to oxygen plasma at a pressure of 20 mTorr for 10 s at room 22 °C.

References

[1] A. Schmitt, M. Mahanthappa, *Computer Code for Materials Scientists: Igor Pro Procedures for Analyzing Dynamic Light Scattering, Rheology, and Synchrotron X-ray Scattering Data, figShare* **2013**.

[2] [a]J. S. Pedersen, M. C. Gerstenberg, *Macromolecules* **1996**, *29*, 1363-1365; [b]J. S. Pedersen, P. Schurtenberger, *Macromolecules* **1996**, *29*, 7602-7612; [c]J. S. Pedersen, *Journal of Applied Crystallography* **2000**, *33*, 637-640.

[3] E. J. Cornel, S. van Meurs, T. Smith, P. S. O'Hora, S. P. Armes, *J. Am. Chem. Soc.* **2018**, *140*, 12980-12988.

[4] M. Semsarilar, V. Ladmiral, A. Blanazs, S. P. Armes, *Langmuir* **2012**, *28*, 914-922.

[5] M. J. Derry, L. A. Fielding, N. J. Warren, C. J. Mable, A. J. Smith, O. O. Mykhaylyk, S. P. Armes, *Chemical Science* **2016**, *7*, 5078-5090.

[6] F. L. Hatton, J. R. Lovett, S. P. Armes, *Polym. Chem.* **2017**, *8*, 4856-4868.

[7] M. J. Rymaruk, S. J. Hunter, C. T. O'Brien, S. L. Brown, C. N. Williams, S. P. Armes, *Macromolecules* **2019**, *52*, 2822-2832.
